# Supplementary material for: Are carbon emissions trading and green financial instruments synergistic? -Comprehensive quantitative research based on content analysis
Source: PLoS One. 2024 Mar 7;19(3):e0298601. doi: 10.1371/journal.pone.0298601 (PMC10919601; doi:10.1371/journal.pone.0298601)
Supplement: S1 Data — (ZIP) [file pone.0298601.s001.zip › Data/地级市Z_F工作报告.zip!/╣π╢1⁄2/╖≡╔╜╩╨/└┤╫╘╣1⁄2╓┌║┼í╛┬φ┐╦╩2╛▌═°í┐_2005.docx]

各位代表：

　　 我代表佛山市人民政府向大会作政府工作报告，请予审议，并请政协各位委员和其他列席人员提出意见。

2004年工作回顾

　　2004年是深化改革、加快发展、狠抓落实的关键一年，是建设产业强市、文化名城和现代化大城市取得新成效的重要一年，也是各项建设全面展开之年。一年来，在省委、省政府和市委的正确领导下，在市人大及其常委会和市政协的监督支持下，市政府坚持以邓小平理论和“三个代表”重要思想为指导，认真贯彻党的十六大、十六届三中、四中全会和胡锦涛总书记视察广东重要讲话精神，树立和落实科学发展观，以发展为第一要务，围绕市委九届三次全会和市十二届人大二次会议提出的“三大目标、十项措施”，抢抓机遇，改革创新，推动经济和社会各项事业全面发展，市十二届人大二次会议确定目标任务基本完成，在建设现代化大城市和宽裕小康社会的发展道路上迈出可喜步伐。

　　 ——经济平稳快速健康发展。据初步统计，2004年全市完成生产总值1653.70亿元，增长16.3%（绝对值按当年价，增长按可比价计，下同）；人均生产总值47500元（按户籍人口计算），增长14.5%。经济运行质量和效益不断提高。地方财政一般预算收入95.29亿元，按可比口径计算增长16.28%；税收总额320.06亿元，增长17.19%；金融部门年末人民币存款余额3258.85亿元，增长11%。

　　 ——产业结构优化调整成效明显。农业结构进一步优化。农业总产值155.81亿元，增长3.5%；农村经济总收入4077.42亿元，增长17.9%。粮食生产有效恢复，农业现代化园区建设进程加快，特色农业、效益农业和农业产业化取得新进展。建设国家级农业龙头企业1家、省级农业龙头企业3家。有效防治高致病性禽流感和重大动物疫病发生，确保畜牧业稳定发展。工业产业整合集群提升。完成工业总产值4058.96亿元，增长20.4%。产业适度重型化、高级化趋势初现，重工业产值增长30.3%，比轻工业增幅高7.3个百分点。高新技术产业发展迅速，高新技术产品产值1002亿元，增长36%。装备工业加快发展，汽车配件等新兴产业呈现蓬勃发展态势，引进一批汽车配件制造企业，形成布局鲜明的汽配产业带。支柱产业竞争力增强，十大支柱产业产值占全市工业总产值的80%以上。名牌带动战略成效显著。新增中国名牌产品15个、广东省名牌产品32个、国家免检产品16个。工业园区和产业基地建设初具规模。顺德科技工业园、南海科技工业园、沧江工业园、禅城高新区以及三水科技工业园产业集群效应显现，顺德区、三水区分别增设国家火炬计划家电产业基地、电子电器产业基地和国家新型显示器件产业园。民营经济发展后劲增强。规模以上民营企业实现工业产值1668.18亿元，增长30.4%，占全市规模以上工业总产值的50.1%，对全市工业总产值增长的贡献率为55.6%，成为我市经济增长的中坚力量。现代物流业发展取得新突破。成功举办佛山（国际）物流合作洽谈会，一批国内外知名物流企业进驻佛山，专业物流基地建设步伐加快，全市物流业增加值276.34亿元，占第三产业增加值的45.1%。商贸流通市场运行良好。引进沃尔玛等国际零售巨头以及国内品牌连锁企业，专业市场、物流配送、会展经济快速发展，金融服务、电子商务、房地产等行业发展势头良好，食品、汽车、石油及制品等消费增长迅速。社会消费品零售总额542.19亿元，增长14.6%。以佛山“新八景”促进历史文化资源开发与旅游产业发展的有机统一，全市旅游总收入89.56亿元，增长33.51%。

　　 ——城镇化和重大基础设施建设步伐加快。城镇化进程加快。编制《佛山市城市发展概念规划》、《佛山市城市总体规划（2004～2020）纲要》和《佛山市市域城镇体系规划》，启动了“2+5”城市组团建设。认真落实省城镇化试点工作，出台《佛山市推进城镇化试点工作的实施意见》，建立城市规划管理新体制，出台了相应管理办法，城镇化试点和城市规划管理各项措施逐步推进。重大基础设施建设全面推进。“一环”城市快速干线等一批交通主干道工程、中心组团新城区、省运会场馆、新闻中心、顺德演艺中心等工程项目建设全面推进。启动三年城乡水利防灾减灾工程建设，按计划完成当年任务。能源建设进程加快。

　　 全年电网建设与改造投资近30亿元，建成投产110千伏及以上工程13项，燃气管网工程及主输气干线开工建设。全社会固定资产投资总额565.40亿元，增长33.4%。认真贯彻落实宏观调控政策。全面清理在建和拟建固定资产投资项目，对不符要求的项目进行停建缓建，完善招商引资项目的行业准入制度。加强土地资源管理，清理整合工业园区，发挥了土地集约利用效能。

——招商引资与区域合作向纵深发展。创新招商引资方式，建立和完善“三大机制一支队伍”，形成灵活高效的引资机制。成功举办2004中国佛山（日本）投资推介会，签约投资总额6.15亿美元。全市合同利用外资18亿美元，实际利用外资17亿美元，分别增长42.2%和38.7%。投资佛山的世界500强跨国公司累计29家，其中新落户9家。佛山海关实行五区海关合一新体制，优化了外向型经济发展环境。外贸出口持续增长，出口主体、出口贸易方式、出口市场和出口商品结构实现“四个优化”。全市完成进出口总值216.9亿美元，其中出口138.3亿美元，分别增长31.7%和35.3%。积极融入广佛经济圈和“泛珠三角”区域合作与发展，加强与粤港澳及全国其它地区的经贸合作，拓宽了资源配置和产业发展空间。加强与吉林延边的经贸合作，并与广西贺州、宁夏吴忠、云南红河洲建立经贸合作关系。

——用“三化”解决“三农”问题实现新突破。制定和落实《关于加快农村工业化城镇化和农业产业化建设的决定》和配套政策文件。统一全市户籍管理；通过建立农村富余劳动力职业培训和就业引导机制，初步建立市、区、镇（街道）三级一体化的农村富余劳动力培训就业服务网络；以基本医疗、基本养老保险补贴和最低生活保障为重点，逐步建立和完善农村居民社会保障体系。全年免征农业税6300多万元，农民负担继续减轻。

——生态环境保护工作稳步推进。全面实施可持续发展生态环境规划纲要，珠江流域、水和大气污染综合整治全力推进。工业污染防治、生态环境保护工作进一步加强，关停一批重污染企业，城镇生活污水处理厂、垃圾处理厂建设步伐加快，治污保洁、电厂脱硫逐步推进。创建国家环保模范城工作全面推进。

——各项改革进一步深化。投融资体制改革迈出新步伐。出台《佛山市推进城市可经营项目投资多元化市场化的指导意见》，整合城市资源，开放公共事业投资领域，推进污水处理、省运会场馆、公共交通、市政建设维护、教育、医疗等城市可经营项目市场化社会化改革，共推出63个项目，投资总额49.2亿元。国有资产管理体制和企业改革取得新进展。进一步理顺国有资产管理体制，企业产权多元化改革步伐加快，佛山市商业银行顺利被兴业银行并购，一批国有企业股权实现转让。全市国有粮食企业改革顺利完成。积极利用资本市场，扶持一批企业完成改制进入上市辅导，协助南海南方包装公司等企业成功上市。社会管理体制改革持续推进。培育和发展各类社会中介组织，整合和规范一批产业性行业协会、商会，发展金融担保机构9家，社会职能作用得到发挥。各区按照“五统一”的原则完成公务用车制度改革。行政管理体制改革稳步推进。经过努力，简政放权后的市、区、镇（园区、街道）三级政府的管理职责和事权已逐步到位，初步实现政府管理重心下移。按省的部署基本完成市和区两级政府机构改革，镇（街道）行政管理体制改革已启动，并镇、并村力度加大。理顺公安管理体制，优化了警力配置，我市被确定为“全国科技强警示范城市”。

　　 ——文化名城建设取得新成效。制订和实施《建设文化名城规划纲要》及配套政策，城市品位进一步提升。整合新闻传媒资源，组建佛山日报传媒集团。成功举办“魅力佛山”琼花粤剧艺术节、武术文化节、中国（石湾）陶艺节等系列活动。引入广东粤剧艺术大剧院，广东粤剧博物馆在佛山挂牌。佛山市民间艺术研究社、佛山市孔雀廊影音电器有限公司被确定为首批国家级文化产业示范基地，南方报业传媒产业基地落户南海，“三水大旗头村保护和利用”项目在首届深圳国际文化产业博览会上正式签约。基层文化建设加快发展，文化设施、重点文物修缮工作按计划推进。对外文化交流进一步拓展，文化市场规范发展。教育事业发展迅速。“以区为主”的教育管理体制进一步落实，教育强区、强镇建设不断加强，全市建设教育强区2个，教育强镇31个。中小学校布局调整力度加大，撤并中小学校112所；有137所中小学（幼儿园）通过省或市一级验收，小学、初中和普通高中的优质学位分别达到56.8%、73.7%和88.2%；加快佛山职工医学院、佛山教育学院并入佛山科技学院的工作步伐，以高等职业教育为重点的大学园区建设稳步推进，基础教育、职业技术教育、高等教育质量和水平进一步提升。科技创新能力得到增强。新增各级工程技术开发中心40家、技术创新示范专业镇5个、高新技术企业78家、省民营科技企业64家，我市被确定为“全国专利工作试点城市”。信息化建设取得新成效，信息化综合指数达78%。人才强市战略全面实施。人才培养、引进和使用配套政策措施进一步完善，高层次人才队伍建设步伐加快，引进博士235名，建立企业博士后工作站11个，在站博士后科研人员43人。卫生、体育事业不断发展。公共卫生体系建设进一步加强，形成了覆盖全市的公共卫生三级网络。卫生防疫预警机制得到完善，初步建立起食品、药品、农产品卫生安全监管新模式，我市6个市场被确定为全省试行市场食品准入制度的试点单位。农村和社区卫生、妇幼保健、科技兴医等工作取得新进展。积极做好省运会各项筹备工作，多元化全民健身服务体系逐步完善，群众性体育活动蓬勃开展，我市荣获“武术之城”称号。

——精神文明建设和社会各项事业再上新台阶。以创建文明城市为动力，以提高人的素质为根本，深入贯彻实施《公民道德建设实施纲要》，广泛开展文明窗口、社区、村镇等群众性精神文明创建活动，市民的思想道德水平、民主法制意识、科学文化素质和城乡文明程度得到提升，未成年人思想道德教育进一步加强。国防动员、民兵预备役和优抚安置工作进一步巩固，荣获双拥模范城全国“五连冠”和广东省“六连冠”。在香港成功举办佛山市荣誉市民授荣仪式，内外同心建设佛山的氛围进一步形成。人口与计划生育工作在巩固中得到强化。人口与计划生育工作稳定健康发展，较好完成省下达的各项人口计划指标。人民生活不断改善。“十项民心工程”深入落实，认真解决困难家庭子女读书、渔民生产生活困难等关系民生热点难点问题，扎实推进农民减负增收、教育扶贫助学、外来工权益保护、全民安居等工作。在全市实行了老年人和残疾人优待办法，着力改善人民生活。全市农村居民人均纯收入7048元，增长6.6%?鸦城市居民人均可支配收入16045元，增长8.2%；城乡居民储蓄存款余额2028.81亿元，比年初增长13.7%。就业和社会保障水平稳步提高。全年安排就业再就业资金6179.04万元，安置就业再就业人员54869人，城镇登记失业率为2.27%。社会保障体系不断完善，社会保险覆盖面进一步扩大，全市参加养老保险126.6万人、增长5.83%；失业保险96.3万人、增长7.53%；医疗保险107.97万人、增长17.22%；工伤保险129.2万人、增长7.16%。城乡居民最低生活保障基本实现“应保尽保”。

——民主法制建设和政府自身建设进一步加强。认真执行市人大及其常委会的决议，坚持向市人大及其常委会报告工作并接受监督。重视和支持人民政协以及各民主党派、工商联履行政治协商、民主监督和参政议政职能。认真办理人大代表议案、建议125件、政协委员提案230件。加强电子政务建设，严格政府采购，完善招投标制度。全面贯彻实施《行政许可法》，落实行政执法责任制，综合行政执法工作顺利推进。政府自身建设得到加强。出台《公务员行为规范》，完善考核制度。充分发挥监察、审计等监督机关的职能作用，设立行政投诉中心，强化行政效能监察作用。落实“为民、务实、清廉”要求和党风廉政建设责任制，市政府向社会公开作出并认真履行四项廉政承诺。落实社会治安综合治理各项措施，推进社会治安防控体系建设。加大对治安重点地区和突出治安问题的整治，坚决扫除“黄赌毒”等社会丑恶现象，保障人民群众安居乐业。认真排查、调处和化解各种矛盾和纠纷，重视做好人民调解和群众来信来访工作，加强政府机关与传播媒体互动机制建设，切实关注民生。推进村（居）务公开，强化了基层民主法制建设。坚持不懈地抓好安全生产，做好全国非公有制中小企业安全生产监督管理试点和推广工作，建立安全生产长效机制，交通、消防和森林防火安全态势平稳。大力整顿和规范市场经济秩序，社会信用体系建设步伐加快。

与此同时，残疾人事业、妇女儿童事业、粮食储备、民族、宗教、外事侨务、对台事务、广播电视、人防、气象、保密、档案、物价、统计、老龄和对口扶贫等各项工作都取得新成绩。

各位代表！过去一年的成绩，是全市人民在党中央、国务院和省委、省政府以及市委的正确领导下，同心同德、奋力拼搏的结果，是市人大及其常委会和市政协监督支持的结果，是中央、省驻佛山单位和社会各界关心支持的结果。在此，我代表佛山市人民政府，向辛勤工作在各条战线的广大干部群众，向给予政府工作大力支持的人大代表和政协委员，向中央、省驻佛山单位，向驻佛山解放军、武警官兵和各民主党派、工商联、人民团体以及社会各界人士致以崇高的敬意！向所有参与、关心和支持佛山建设的港澳台同胞、海外侨胞和国际友人表示衷心的感谢！

在肯定成绩的同时，我们也清醒地看到，我市经济和社会发展仍然存在不少困难和问题，主要表现在：经济结构依然不合理，产业发展后劲不强；科技自主创新能力有待加强；经济社会健康发展仍面临许多体制机制性障碍；发展中的资源约束比较突出；“三农”问题仍有不少深层次矛盾需要解决；城市建设滞后于经济发展，现代化大城市意识有待提高，生态环境有待进一步改善；政府治政理念和职能转变还有不适应的地方等等。对这些问题，我们一定要增强忧患意识和紧迫感、使命感，采取有力措施，认真加以解决。

2005年的工作安排

2005年是全面实现“十五”计划目标、衔接“十一五”计划发展的重要一年，是贯彻落实科学发展观，进一步推进建设产业强市、文化名城和现代化大城市取得成效的重要一年。进入新一年，世界经济将继续保持较快增长，国内发展环境进一步改善，我市经济发展处于新一轮上升周期，各区积极性、主动性和创造性充分调动起来，各项规划的实施和各项重点工程建设全面推进，全市形成建设发展新局面，发展动力继续增强，发展空间不断拓展。但是，世界经济发展也还存在不确定因素，国内经济发展的生产要素紧张状况短期内不会改变，周边兄弟城市加快发展态势逼人，我市发展面临更大竞争压力。对此，我们必须认清形势，增强信心，抢抓机遇，以科学发展观谋划发展新思路，实现经济社会全面协调可持续发展。

2005年政府工作的指导思想是：以邓小平理论和“三个代表”重要思想为指导，全面贯彻党的十六大、十六届三中、四中全会、中央经济工作会议和胡锦涛总书记视察广东重要讲话精神以及省委九届六次全会精神，认真落实市委九届六次、七次全会的工作部署，围绕建设产业强市、文化名城和现代化大城市的目标，以科学发展观统领经济社会发展全局，坚持以发展为第一要务，推动产业结构优化升级，提升经济增长质量和水平；大力发展文化事业和文化产业，促进文化与经济融合，加快社会事业发展，建设文化名城；加快现代化大城市建设，努力营造良好的创业环境和生活环境；加强民主法治建设，促进依法行政，建设文明法治社会；切实解决人民群众生产生活问题，努力构建稳定和谐、富裕安康的新佛山。

今年我市经济社会发展主要预期目标：生产总值增长12%；全社会固定资产投资增长12%；社会消费品零售总额增长13%；地方财政一般预算收入增长10%；出口总值增长15%；实际利用外商直接投资增长10%；城市居民人均可支配收入增长7%；农村居民人均纯收入增长5%；居民消费价格总指数上升3.8%左右；城镇登记失业率控制在3.5%以内；人口自然增长率控制在5.9‰以下。

为实现上述目标任务，必须着重抓好以下十项工作：

　　 一、着力优化产业结构，提高经济增长质量和效益

进一步优化产业结构，提升产业集群发展水平。落实产业发展规划，优化产业结构和产业布局，强化以汽车配件和精密制造为重点的装备制造业发展规划和政策引导，做大做强装备制造业，使其成为新的经济增长点。继续贯彻落实国家宏观调控政策，高标准建设国家级、省级高新技术产业开发园和产业基地，加快形成科技成果孵化体系；提高产业集群的核心竞争力，加快产业技术研发、技术信息服务、产品检验检测、标准质量认证等中心建立，构建产业发展技术支撑平台。增强产业集群内在凝聚力，发挥核心企业辐射和纽带作用，带动上下游企业集聚，培育一批大企业，建设一批竞争力强的行业。发挥专业镇在产业集群发展中的基础作用，提升专业镇发展水平。

　　 加快发展高新技术产业，改造提升传统产业。大力发展电子信息、新材料、光机电一体化、生物医药等高新技术产业；进一步推动科技成果转化，提升产业竞争力；运用高新技术和先进适用技术改造提升纺织服装、家电、金属制品及加工、建筑材料等传统产业。抓好现有企业技术改造和增资扩产，做强做大一批有自主知识产权、有国际竞争力的龙头企业，推动企业向集约化、规模化发展。实施名牌带动战略，培育一批高新技术产业的知名品牌和龙头企业，使之成为产业强市的主力军。

　　 大力发展现代流通业，提高服务业发展质量和水平。充分发挥佛山制造业实力雄厚、区位和交通便利等优势，加快服务业发展。抓好物流洽谈会签约项目的落实，推进物流中心和物流基地的加快发展。制定发展扶持政策，加快传统物流业的有效整合和改造提升，支持国际物流基地建设，大力培育第三方物流。积极引进国内外知名第三方物流企业，实现物流社会化、集约化。加强与广州新白云机场、南沙航运港的物流对接，建立顺畅的国内外物流通道。积极推动连锁经营等新型业态的发展，培育和提升一批具有国际化水平的中高级批发市场。积极发展商贸、饮食、旅游、会展、信息、金融、中介等为生产和生活服务的现代服务业，大力引进外资尤其是港资进入服务领域，通过结构调整努力扩大服务业增长空间，提高服务业发展质量和水平，促进二、三产业协调发展。

　　 推动区域产业结构调整优化，做优做强东南部，加快发展西北部。统筹东南部和西北部协调发展，推动东南部地区通过承接新一轮国际产业转移和传统产业的转移提升，加快发展高新技术产业和密集型产业，提高产业发展层次，实现快速提升；推动西北部地区利用后发优势，积极承接产业转移，做优做强产业，实现跨越式发展。同时，积极融入“广佛经济圈”和“粤港澳”经济区域，参与泛珠三角区域和国内外其它地区的合作与发展，承接世界产业转移，在合作中拓展、壮大和巩固产业发展优势。

　　 二、抓好招商引资和民营经济工作，推动内外源经济协调发展

　　 创新招商引资方式，提高招商引资的质量和水平。进一步优化投资营商环境，增强对外资的吸引力。高度重视引资项目的规模与质量，重点引进世界500强企业、跨国公司和国际大企业的投资，引进跨国公司、营运中心、研发中心和跨国采购机构，引进高新技术项目和有利于延长产业链、提升产业配套能力的大项目，促进产业结构优化升级和集约发展。健全和完善招商引资的协调、协作机制，落实招商引资责任制，优化跟踪服务制度。在推行以商引商、中介招商、小分队招商方式的前提下，积极推进产业招商、资本并购、BOT等招商引资方式。不断优化招商引资结构，积极引进北美、欧盟及日韩等地区资本，引入汽配、家电、新材料和精密制造等行业。鼓励和支持外资企业增资扩产，提升外资企业发展水平。继续抓好已签约项目跟进落实，确保履约率、开工率、投产率的提高。

　　 积极拓展国际市场，保持外贸出口增长态势。巩固港澳、欧美日等传统市场，大力拓展非洲、南美、东盟、俄罗斯等新兴市场，努力增加出口。充分利用我市电子信息、家电、家具、纺织等产业优势，实施品牌带动出口战略，提高出口商品和服务的技术含量和附加值。贯彻落实新的外贸法和出口扶持政策，积极引导和扶持企业参与国际性的博览会、专业展销会，推进出口主体多元化。合理规划口岸布局，推进电子口岸建设。抓紧做好入世后过渡期的各项工作，充分运用WTO规则，做好产业损害预警和应对国外贸易技术壁垒工作，建立和完善重点出口商品的反倾销预警分析系统，提高出口企业应对反倾销能力。

　　 完善促进民营经济发展的政策措施，提升民营企业整体素质和竞争力。全面落实《中小企业促进法》，抓好扶持政策的落实，加快信用担保等服务体系建立，为民营经济发展营造更好的环境。抓好美的集团发展模式推广，推动民营企业与外资结合，与国际公司合资合作。抓好西樵纺织产业技术升级经验的推广，鼓励民营企业技术创新产品换代。引导和支持民营企业进入资本市场，转换机制，提高发展素质。放宽市场准入和投资领域，引导民营企业参与国有企业改革，鼓励和支持外地民营企业在我市投资发展。鼓励和引导民营企业创立品牌、提升核心竞争力。

　　 三、以城乡一元发展为目标，加快城市化进程

　　 加快推进“城乡一元结构”发展，解决好城市化进程中的矛盾和问题。进一步加大“农村工作五十条”和配套文件的落实力度，引导和推动农村居民进厂务工、进城创业、融入城市。加快出台农村经济产权改革、“村改居”等方面政策文件，解决好城乡统筹发展中的深层次矛盾和问题，推进“三化”解决“三农”问题实现新突破。加快中心镇的建设步伐，继续推进“并村、并镇”和“村改居”、“迁村并点”工作。大力推进农民公寓建设，加快“城中村”改造，把“城中村”改造为现代文明社区。

　　 大力调整优化农业结构，提高现代化水平。进一步调整优化农业结构，实施科技兴农战略，推广农业新品种、新技术，提高农业科技贡献率，加快发展高质农业、绿色农业、创汇农业、都市农业和观光农业。保障粮食种植面积，抓好粮食储备工作。落实《佛山市建设农业现代化园区的实施意见》，抓好农业园区的规划建设，以农业园区、龙头企业和规模化、专业化生产基地为依托，大力发展农民专业合作经济组织，提高农业生产规模化、组织化和产业化程度。大力发展农产品流通和加工，提高农产品附加值和经济效益。同时，加快农产品市场准入步伐，抓好农产品质量安全管理体系建设，确保人民群众吃上放心农产品。

　　 四、以规划为龙头，加快城市基础设施和生态环境建设，提升城市综合竞争力

　　 强化规划龙头作用，提升现代化大城市建设水平。突出规划在城市建设中的龙头作用，以加快城市基础设施和生态环境建设，提升城市综合竞争力为着眼点，加大规划投入，完成城市总体规划及一系列专项规划，强化规划实施手段。

　　 按照“统一规划、统一标准、统一安排、属地实施”原则，加快推进组团城市建设。发挥组团城市建设领导协调机制作用，抓好中心组团建设的引领和示范带动，加快推进其它城市组团建设。加快各组团资源优化配置，推动体制、基础设施、市政道路和人文环境整合，促进人口、资源、公共服务设施和土地利用向各组团集中，加快各组团之间联接，提升各组团在支撑整个组团式大城市建设中的战略地位和推动作用。要把加快旧城区改造作为组团城市建设的重要内容，做好规划，逐步实施，改善城市环境，提升城市形象。

　　 加快以“一环”为重点的交通路网建设，完善城市综合交通体系。全力推进“一环”城市快速干线等主干道路第一批项目的建设，加快广明高速公路建设，积极配合省做好珠二环等一批途经佛山高速公路和广佛地铁、广珠城际轨道交通的建设。充分利用江河资源，搞好水运交通规划建设。抓好以中心组团为重点的“2+5”组团道路建设，完善城市组团公共交通网络系统。通过地面、地下和水上的交通建设，实现与广州及其他周边城市在交通基础设施上的衔接。合理调整完善机动车路桥年次票征费方案，巩固交通建设投资体制改革成果。

　　 加快中心组团新城区和体育、文化场馆建设，提升城市形象。切实抓好中心组团新城区和“岭南明珠”体育场馆、“世纪莲”体育中心、新闻中心、东平大桥等各项工程项目建设，为明年省运会举办打下坚实基础。抓好市容市貌和主要道路两旁整治，加快顺德演艺中心、亚洲艺术公园等项目建设，以崭新面貌迎接第七届亚洲艺术节和亚洲文化部长论坛在我市举行。

　　 加强电源和电网建设，努力解决经济社会发展瓶颈问题。认真落实电力建设规划，并将电力规划纳入城市总体规划。认真落实电网建设绿色通道和电力设施用地补偿方案等有关规定，完善电网结构。加大电源建设力度，争取顺德德胜电厂二期和南海A厂二期、沙口电厂扩建工程早日开工建设和完工，提高供电能力。

　　 加强生态环境建设，积极创建国家环保模范城市和国家卫生城市。落实《佛山市可持续发展的生态环境规划纲要》，全面推进创建国家环保模范城和国家卫生城各项工作，以“创模”和“创卫”为目标和动力，推进珠江综合整治和治污保洁工程，加大污染源治理力度，加快环境基础设施建设和城市绿化系统建设。推广顺德经验，加大内河涌整治力度。加强城市饮用水源保护，加强生态示范区、生态示范镇和绿色社区创建工作。推行清洁生产，大力发展循环经济。认真抓好城市大气污染和机动车尾气、重点行业二氧化硫及粉尘污染的整治，关停一批治理无望的污染企业。加大环境卫生整治力度，全面提升城乡卫生水平。

　　 五、进一步深化各项改革，增强经济发展活力

　　 积极推进产权制度改革。优化公有经济布局和结构，加大产权改革与重组力度，多种形式推进产权多元化改造，构建完善的法人治理结构和健全的现代企业制度。通过对国有企业优化重组，着力发展壮大关系民生的产业和企业。推进企业资本运营，鼓励民资和外资参与国有企业改革改组改造。进一步完善公有资产管理体制，建立管人管事和管资产相结合的管理体制，完善公有资产监管制度，实现公有资产保值增值。

　　 深化投融资体制改革。按照开放、让利、公平的原则，抓好交通、第二批生活污水处理、固体工业废物处理、水利工程、市政工程、文化、旅游等一批城市可经营项目市场化运作。鼓励社会力量、民营资本进入市政基础设施、文化、教育、医疗、体育等领域。探索将历史文化资源保护与利用、旅游资源的开发向社会资本开放。发挥金融支持经济发展的作用，防范金融风险，加强信用佛山建设。积极拓宽融资渠道，加大利用资本市场力度，支持和鼓励各类企业改制上市，壮大企业发展规模。

　　 加大现代市场体系建设。培育和发展技术、土地、劳动力等要素市场，规范发展有形建筑市场和房地产市场交易行为。抓好市场经济秩序整治，强化对各类市场监管，优化市场环境，把物价上涨幅度控制在预期目标之内。培育发展各类社会中介机构和行业协会，建立信息网络系统和市场供求信息合作机制，完善市场服务体系。

　　 推进土地管理制度改革。贯彻落实《国务院关于深化改革严格土地管理的决定》，切实保护基本农田，挖掘土地潜力，保障重点工程和产业发展用地。深化征地制度改革，完善征地补偿安置制度，保护被征地农民合法权益。按省部署，抓好国土资源行政管理体制改革。

　　 六、以办好亚洲艺术节为契机，加快文化名城建设

　　 全力办好亚洲艺术节，提升佛山城市形象。要以高度的政治责任感和主人翁精神，圆满承办好第七届亚洲艺术节和亚洲文化部长论坛。同时，精心办好首届“广东省粤剧艺术琼花奖”和“中国广东国际音乐夏令营”等大型文化活动，继续举办“魅力佛山”系列文化艺术活动，进一步提升佛山文化形象和辐射力。

　　 加快发展文化事业和产业。积极稳妥推进文化管理体制改革，抓好文化、广播电视和新闻出版资源整合。落实好促进文化产业发展政策，打造文化产业品牌。加快发展数字广播电视电影和创意产业等新兴文化产业。鼓励发展民营文化企业，加快形成多元化投资、全方位发展的文化产业体系。加快文化设施建设，加强基层文化发展，强化文化市场管理。

　　 进一步整合开发利用历史文化资源。加强对佛山深厚历史人文资源的保护和开发利用，弘扬陶艺、民间艺术、粤剧和武术等传统文化，加强对佛山祖庙、大旗头村等重要文化遗产以及优秀民俗民间文化的保护和利用。加大佛山各历史文化资源向市外、省外的宣传、推介力度，扩大我市文化旅游资源的影响。充分利用顺德“中国厨师之乡”品牌，发扬光大优秀传统饮食文化。

　　 努力构建和完善科技创新体系。创新科技体制，加强研究开发院、科技创新中心和企业研发中心建设，吸引世界500强企业和境外有实力的公司到佛山设立研发机构，不断提高企业创新能力和竞争实力。扶持电子信息、绿色智能家电、光机电一体化、新材料、生物技术等领域的科技攻关，力争取得一批具有自主知识产权的科技成果。加强产学研合作，构建以科研院所和大专院校为重点的知识创新体系，提高自主创新能力。加强知识产权保护，为科技创新和专利技术实施提供保障。拓展信息技术在经济社会领域应用范围，全面提升国民经济和社会信息化水平。

　　 大力发展教育事业。加快推进教育强区、强镇建设步伐，力争建成广东省教育强市。完善九年义务教育“以区为主”的教育管理体制，推进中小学布局调整，加大等级学校建设力度，努力完成全市12所国家级示范性普通高中和10所国家级重点中等职业技术学校的建设任务，不断提高中小学优质学位，逐步扩大全市高中阶段学校统招规模。加强以高等职业教育为重点的大学园区建设，构建职业教育、高级技工教育、技师教育的职业教育体系。创造条件，努力实现市、区属的大专院校面向全市招生，并拿出部分名额向省内外招生。高度重视外来人口教育以及外来工子女入学问题。积极扶持民办教育发展，规范民办教育管理。深化教育改革，全面推进素质教育，以信息化带动教育现代化，促进依法治教，切实保障教师合法权益，建设一支适应教育现代化需要的教师队伍。

　　 积极推进人才资源开发。落实各项政策措施，构建有利于人才发展的公共服务平台，营造有利于各类人才脱颖而出和干事创业的社会环境。以能力建设为中心，切实抓好党政、企业经营管理、专业技术、高级技能等4支人才队伍的建设，加快人才市场、企业博士后工作站、留学人员创业园建设，加强珠三角、泛珠三角及国（境）外等人才合作交流和海外智力引进。建立完善科学的人才评价指标体系和考核机制。

　　 健全完善公共医疗卫生体系。健全公共卫生突发事件应急处置机制，建立和完善疾病预防控制、医疗救治和卫生执法监督等体系。加强公共卫生体系基础设施建设，建设公共卫生三级网络。全面推进镇村医疗卫生管理一体化，完善社区卫生服务网络。继续抓好非典、高致病性禽流感、结核病、艾滋病等重点传染病预防控制工作。

　　 加快发展体育事业。全面推进第十二届省运会各项筹备工作，营造举办省运会的良好环境。充实体育人才资源，提升竞技体育水平。构建多元化全民健身服务体系，推动群众性体育活动深入开展。

　　 推动人口与计划生育和其它社会各项事业发展。加大依法行政、优质服务、综合改革力度，提高流动人口计划生育管理和服务水平，完善计划生育利益导向机制。加快妇女儿童事业发展，重视做好统计、外事侨务、台务、人防、宗教、档案、方志和老龄等各项工作。

　　 七、依法征税理财，努力增收节支

　　 加强税收征管，积极组织收入。落实稳健财政政策，促进发展，优化结构，积极培植财源，确保财政稳步增长。加强依法征税，严厉打击偷税漏税等违法犯罪活动。

　　 规范财政管理，完善支出机制。厉行节约，反对铺张浪费，严格支出管理，集中财力确保社会保障、就业再就业、教育、科技、人才等重点支出需要。加大对解决“三农”问题以及交通、城建、体育场馆等基础设施建设的投入，建立重大基础设施建设项目还本付息专项资金机制。

　　 强化财政监督，促进依法理财。进一步加强依法理财观念，强化财政监督机制。严格部门预算，提高预算编制的质量和水平。围绕公共财政体系建设，深化财政体制改革。积极推进国库集中支付改革，进一步强化社保费征缴和规范非税收入管理，切实做到应收尽收。进一步拓展范围，加强和落实“收支两条线”管理工作，积极推动政府采购制度向纵深发展，充分利用信息化手段实现市、区、镇三级资源共享，提高政府采购效率。积极稳妥推进财政支出绩效评价，逐步推行绩效预算的试点工作。

　　 八、认真解决群众生产生活实际困难，实现富裕安康

　　 健全和完善一体化的就业机制。落实各项就业政策，全面实施就业再就业工作目标责任制，积极改善就业环境，完善就业服务和职业培训体系。加快城乡就业一体化进程，按照“劳动就业与技能培训城乡统筹，劳动力市场城乡统一；就业和再就业服务城乡平等，就业资格城乡平等，就业技能培训城乡平等；劳动保障待遇城乡逐步接轨”的“两统三平等一接轨”的思路，建立和完善农村居民就业培训机构、机制、阵地和培训就业政策，从组织、制度和政策上建立起农村富余劳动力培训和就业的长效机制。鼓励社会力量参与农民就业培训，通过灵活多样的培训途径，提高农民创业能力和就业实现率；对吸纳当地农民就业的企业加大支持力度，发挥镇、村（居）在劳动就业服务中的主力军作用，帮助全征地农民实现充分就业。同时，加快企业退休人员社区化管理步伐。

　　 加快建设与经济发展水平相适应的社会保障体系。实施“两个确保”，完善城镇职工养老、失业、医疗、工伤、生育等基本保障制度及住房分配货币化、住房公积金制度，进一步扩大覆盖面。推进社会保险市级统筹。建立和完善以基本医疗保险、基本养老保险补贴、最低生活保障为重点的农村居民社会保障体系，提高基本保障水平。对进厂务工的农村劳动力，实行城镇职工的社会保障管理，逐步实行与城镇职工统一的社会保障服务。

　　 不断改善人民群众生活。扎实推进“十项民心工程”，帮助困难群众解决子女入学难、看病难、打官司难、住房难等“四难”问题。推进城乡水利防灾减灾工程建设，确保完成工程总量45%的年度建设任务。关心残疾人就业、基本生活和福利事业。继续抓好渔民生产生活困难问题的解决，妥善解决好优抚对象、企业军转干部、伤残军人及困难群众生活保障问题。

　　 九、营造文明法治安全社会环境，努力构建和谐佛山

　　 加强精神文明建设。坚持依法治市和以德治市相结合，深入开展“爱国、守法、诚信、知礼”现代公民教育和法制教育，提高广大市民道德法律素质。加强和改进大学生和未成年人思想道德建设，努力构建学校、家庭、社区和传媒“四位一体”的教育体系。以行业、社区、街道、乡村为重点，深入开展群众性精神文明创建活动，努力实现文明城市目标。加强国防动员和全民国防教育，抓好民兵、预备役建设，积极开展双拥工作，争创双拥模范城全国“六连冠”和全省“七连冠”。

　　 推进民主法制建设。坚持向人大及其常委会报告工作。自觉接受人大及其常委会的法律和工作监督，自觉接受人民政协的民主监督，高度重视并认真办理人大代表、政协委员的议案、建议和提案。认真听取民主党派、工商联、无党派人士、各人民团体和民间组织的意见和建议。加强公众参与、专家论证和决策机构决定的制度和机制建设，完善重大事项、重大决策、重大规划和重大政策等征询意见和社会讨论、听证、公示制度，推进决策科学化、民主化和程序化。同时，进一步加强社区基层组织建设和民主法制建设。

　　 努力构建和谐社会。整合社会资源，推进社会保障和救济、社会治安防控、社会公共安全、抗击自然灾害、防范和处置群体性事件等网络以及以110为平台的公共安全应急处置中心的建设。加强社会治安综合治理，依法打击各类违法犯罪活动，扫除“黄、赌、毒”等社会丑恶现象和各类黑恶势力。做好城市生活无着的流浪乞讨人员的救助管理和刑释解教人员安置帮教工作。总结推广我市非公有制中小企业安全生产试点经验做法，高度重视安全生产，加大交通、生产、消防、治安、公共卫生等安全事故防范和处置力度，强化食品、药品安全监督管理，建立健全防范化解重特大安全事故长效机制。高度重视人民来信来访，及时处理群众反映的突出问题。健全矛盾排查调处机制，充分发挥人民调解、行政调解的综合作用，努力把人民内部矛盾和不稳定因素解决在基层，化解在萌芽状态。提高外来务工人员生活水平，加大劳动保障监察力度，积极协调好劳动关系，建立健全工资支付监控机制，保障外来务工人员等所有劳动者合法权益。

　　 十、求真务实，努力建设廉洁高效的服务型政府

　　 切实履行政府职责，狠抓各项工作落实。深化行政体制改革，推进政府管理创新，全面履行政府职能。在搞好经济调节、加强市场监管的同时，切实加强政府的社会管理和公共服务职能，建设服务型政府。进一步推进政务公开和电子政务建设，完善“佛山市现行文件网上查询中心”，尽快实现市和区可公开政务文件的共享和实时查询，提高政府工作透明度，提高政府公信力。强化各级政府及其工作人员的服务意识，更好地为发展服务、为基层服务、为群众服务。

　　 加强政风建设，确保政令畅通。按照执政为民要求，弘扬求真务实精神，进一步改进工作作风，加强调查研究，着力解决改革发展过程中的实际问题。积极推进行政效能监察，继续加强督查督办工作，确保政令畅通、确保各项重点工作落实到位。开展“理想、责任、能力、形象”教育活动，配合《公务员法》的实施，努力建设一支政治坚定、业务精通、清正廉洁、作风优良的公务员队伍。

　　 加强法制建设，确保依法行政。认真贯彻落实《行政许可法》和国务院《全面推进依法行政实施纲要》，严格按照法定权限和程序行使权力，履行职责，规范行为。积极推进新一轮审批制度改革，进一步精简审批事项，提高审批效率，实现五区审批业务信息共享互动，推进市、区两级行政管理体制创新。健全和完善行政执法责任制和过错追究制，加强行政执法监督，促进严格、公正和文明执法。巩固我市所有公路基本无“三乱”的成果。搞好综合行政执法试点，努力提高城市综合执法水平。

　　 加强廉政建设，确保为政清廉。深入开展“为民、务实、清廉”教育，严格执行“四项廉政承诺”和“六不许”。加强行政监督，切实抓好政府系统党风廉政建设。依法加强和完善招投标制度和政府采购工作，加大对干部选拔任用监督及政府领导干部经济责任审计力度。强化财政资金的绩效审计，扩大绩效审计范围。深入持久地开展反腐败斗争，坚持教育自律与制度机制建设相结合，从源头上防止腐败，坚决纠正损害群众利益的不正之风，依法查处大案要案，严惩腐败分子，树立清正廉明、干净干事的政府形象。

　　 与此同时，按照科学发展观要求，认真做好“十一五”规划编制工作。

　　 各位代表！我们正处在全面建设产业强市、文化名城和现代化大城市的重要时期，任务艰巨而光荣。让我们高举邓小平理论伟大旗帜，全面贯彻“三个代表”重要思想和科学发展观要求，在省委、省政府和市委的正确领导下，与时俱进，振奋精神，励精图治，众志成城，在新起点上努力开创佛山改革开放和现代化建设新局面！
